# Pilot Investigation on p75ICD Expression in Laryngeal Squamous Cell Carcinoma

**DOI:** 10.3390/cancers14112622

**Published:** 2022-05-25

**Authors:** Viviana Triaca, Elena Fico, Pamela Rosso, Massimo Ralli, Alessandro Corsi, Cinzia Severini, Alvaro Crevenna, Enzo Agostinelli, Emma Rullo, Mara Riminucci, Andrea Colizza, Antonella Polimeni, Antonio Greco, Paola Tirassa

**Affiliations:** 1 Institute of Biochemistry and Cell Biology, National Research Council (CNR), International Campus A. Buzzati-Traverso, Monterotondo Scalo, 00015 Rome, Italy; 2 Department of Sense Organs, Institute of Biochemistry and Cell Biology, National Research Council (CNR), University of Rome La Sapienza, 00185 Rome, Italy; elena.fico@ibbc.cnr.it (E.F.); pamela.rosso@ibbc.cnr.it (P.R.); cinzia.severini@cnr.it (C.S.); 3 Department of Sense Organs, University of Rome La Sapienza, 00185 Rome, Italy; massimo.ralli@uniroma1.it (M.R.); enzo.agostinelli@uniroma1.it (E.A.); andrea.colizza@uniroma1.it (A.C.); antonio.greco@uniroma1.it (A.G.); 4 Department of Molecular Medicine, University of Rome La Sapienza, 00185 Rome, Italy; alessandro.corsi@uniroma1.it (A.C.); emma.rullo@uniroma1.it (E.R.); mara.riminucci@uniroma1.it (M.R.); 5 Epigenetics and Neurobiology Unit, EMBL Rome, International Campus A. Buzzati-Traverso, Monterotondo Scalo, 00015 Rome, Italy; alvaro.crevenna@embl.it; 6 Department of Oral and Maxillo Facial Sciences, University of Rome La Sapienza, 00185 Rome, Italy; antonella.polimeni@uniroma1.it

**Keywords:** LSCC, p75NTR, p75ICD, ABCG2, CSCs, tumor invasion

## Abstract

**Simple Summary:**

Laryngeal Squamous Cell Carcinoma (LSCC) is a squamous cancer with 2.4% new diagnoses each year, accounting for 25% of head and neck cancers, and has a high mortality rate. A deeper understanding of key mechanisms and knowledge of the putative target molecules of theranostic relevance are required. The receptor for neurotrophins p75NTR has been shown to be highly expressed in cancer stem cells (CSCs) of squamous epithelia, in LSCC as well as in other cancers. However, whether its cleavage product p75ICD expression, known to finely regulate the survival/death balance in neurons, is also a master regulator in cancer and LSCC in particular has not been directly addressed so far. To resolve this question, we performed a preliminary study using a limited number of LSCC specimens and studied p75ICD presence and expression pattern in LSCC specimens, showing that p75ICD may be a promising target in LSCC, requiring further investigation.

**Abstract:**

We investigated the p75 Neurotrophin Receptor (p75NTR) expression and cleavage product p75NTR Intracellular Domain (p75ICD) as potential oncogenic and metastatic markers in human Laryngeal Squamous Cell Carcinoma (LSCC). p75NTR is highly expressed in Cancer Stem Cells (CSCs) of the laryngeal epithelia and it has been proposed as a marker for stemness, cell migration, and chemo-resistance in different squamous carcinomas. To investigate the clinical significance of p75NTR cleavage products in solid tumors, full-length and cleaved p75NTR expression was analyzed in laryngeal primary tumors from different-stage LSCC patients, diagnosed at the Policlinico Umberto I Hospital. Molecular and histological techniques were used to detect the expressions of p75NTR and p75ICD, and ATP Binding Cassette Subfamily G Member 2 (ABCG2), a CSC marker. We found regulated p75NTR cleavage during squamous epithelial tumor progression and tissue invasion. Our preliminary investigation suggests p75ICD expression and localization as possible features of tumorigenesis and metastaticity. Its co-localization with ABCG2 in squamous cells in the parenchyma invaded by the tumor formation allows us to hypothesize p75NTR and p75ICD roles in tumor invasion and CSC spreading in LSCC patients. These data might represent a starting point for a comprehensive analysis of p75NTR cleavage and of its clinical relevance as a potential molecular LSCC signature, possibly helping diagnosis, and improving prognosis and personalized therapy.

## 1. Introduction

Head and Neck Squamous Cell Carcinoma (HNSCC) is the sixth-most-common cancer worldwide, and Laryngeal Squamous Cell Carcinoma (LSCC) accounts for approximately 10% of all HNSCCs [1].

Despite recent progress in the treatment of HNSCC, including surgery, radiotherapy, and medication-based therapy (chemo, targeted, and immune therapy), a major impact on the survival rate is still lacking and it has remained substantially unchanged over the last 30 years. The general survival rate for LSCC is 61% at 5 years, but, analyzing by tumor stage, the cure rates increase up to 80–90% in case of limited disease (T1 and T2). In case of local advanced diseases (T3 and T4) or regional lymph node involvement, the survival rate is <50% at 5 years [2].

In line with this, there is an unmet medical need for innovative treatment strategies, which are only possible through the investigation of the molecular mechanisms underlying LSCC development and progression, and the discovery of targetable signaling. In particular, characterization at the cellular and molecular levels of chemotherapy-resistance and invasion key players will be critical for the design of new drugs, as well as for the improvement of the clinical management of LSCC patients.

Accumulating evidence over the last decade pinpointed the p75 Neurotrophin Receptor (p75NTR) as a potential malignant marker in cancer. For example, p75NTR has been implicated in melanoma [3,4,5,6], and its expression has been demonstrated in several non-neuronal cancers, such as Schwann cell tumors, ganglioneuroma, granular cell tumors, and malignant peripheral nerve sheath tumors [7]. The expression of p75NTR is also associated with increased cell proliferation in tongue squamous cell carcinoma [8,9], while the antibody to p75NTR results in decreased HNSCC formation in vivo [10]. Moreover, the anti-proliferative effect of p75NTR knockdown in cancer cells has been found to be mediated by the regulation of the p53 tumor suppression [11], which has been recently reported as an independent prognostic factor in HNSCC patients [12], further supporting the role of p75NTR in the progression of squamous carcinomas.

However, the described biological significance of p75NTR modulation may vary in different types of tumors, as it exerts malignant effects, such as the invasion depth of the cancerous tissue and lymph node metastasis in esophageal cancer [13]. On the other hand, p75NTR can induce pro-apoptotic pathways in digestive cancers, such as hepatic and gastric carcinoma. p75NTR expression is deeply downregulated in gastric cancer, and its reactivation is able to induce the apoptotic process by arresting their cell cycle progression in vitro [14].

In prostatic tissues, p75NTR is absent in hyperplastic benign epithelial cells, and it is specifically overexpressed in prostatic cancer cells, where it correlates with high-risk tumors with an unfavorable prognosis (Gleason score > 7) [15]. Moreover, p75NTR has been studied in LSCC, and its expression and localization have been suggested to correlate with malignant transformation and tumor proliferation [16].

Of interest for the present study, p75NTR may undergo regulated cleavage, generating intracellular fragments able to translocate to the nucleus harboring specific signaling activity, as already demonstrated for the pro-apoptotic activity of the p75NTR Intracellular Domain (p75ICD), released upon the γ- and α- secretases’ sequential cleavages of the p75NTR in neurodegenerating neurons of Alzheimer’s Disease-affected brains [8].

p75NTR-regulated cleavage has been hypothesized to also occur in some cancer types and in tumor cellular models. For example, p75NTR enzymatic maturation has been shown to be critical for glioma invasion and spreading [3].

However, direct proof of the regulated cleavage of p75NTR leading to the generation of p75ICD in cancer cells is still lacking in most malignant tumors, including LSCC.

Here, we used the specific anti-p75ICD antibody, which does not cross react with the p75NTR Full Length (p75NTR FL) molecule, to investigate its expression and localization in primitive LSCC and its nodal metastasis in a small group of patients representative of the diverse stages of LSCC.

Furthermore, since p75NTR is also present in Cancer Stem Cells (CSCs) and it is co-expressed with stemness markers, such as CD133 in LSCC [16], and with adenosine triphosphate (ATP)-binding cassette efflux transporter G2 (ABCG2) in tumors involving different organs, as well as in primitive hepatic and prostatic cancers [17], the possibility that p75NTR might be a novel CSC marker of LSCC has been also explored. The expression and localization of ABCG2, which has been associated with invasion, metastasis, and resistance to chemotherapy in squamous carcinoma and laryngeal carcinoma [18], and is an unfavorable prognostic factor in HNSCC [17], were evaluated by us to investigate the cell-type-specific localization of p75NTR and p75ICD in CSCs in LSCC.

The present study firstly detected the expression pattern of p75ICD and compared it to the p75NTR FL in LSCC, and revealed that p75ICD was particularly upregulated in neoplastic squamous cells expressing ABCG2 and was strongly associated with tumor progression. Moreover, p75ICD expression was cytosolic in stages II-III and became nuclear in neoplastic epithelia and in ABCG2-positive CSCs.

Although these results are arising from pilot study, and therefore need to be further explored in a larger patient group, our findings indicate that p75ICD generation by p75NTR-regulated cleavage might represent a relevant marker of tumor invasion and metastasis in LSCC, and support a role for p75NTR/p75ICD in the regulation of CSC proliferation.

## 2. Materials and Method

### 2.1. Tissue Specimen Collection

We evaluated laryngeal specimens from LSCC surgically removed at the Otolaryngology Unit, DAI Testa-Collo, Policlinico Umberto I University Hospital, 00161 Rome, Italy, from April 2016 to April 2018. All patients included in the study were affected by LSCC and subjected to open partial horizontal laryngectomy or total laryngectomy. Specimens were processed and evaluated at the Section of Pathology of the same hospital, where a definitive histological diagnosis was provided.

The inclusion criterion was confirmed laryngeal cancer treated with surgical therapy. The exclusion criteria were previous radiation therapy on the head and neck region and neoadjuvant therapy. All patients gave written informed consent for inclusion in the study. The use of these clinical materials was approved by the institutional ethics committees (RIF. 6129).

The stage of LSCC was classified according to the 8th edition of the American Joint Committee on Cancer (AJCC, 8th Edition). Relapse-free survival was defined as the interval between the date of surgery and the date of recurrence or last follow-up available.

The study was performed on a total of 8 LSCC patients (absence of residual cancer both macroscopically and histologically in all cases). In Table 1, the pathological TNM (pTNM) stage from the Classification of Malignant Tumor, AJCC stage, and G (Grading) of each patient enrolled are reported. Histopathological evaluation revealed all tumors to be squamocellular carcinoma. One case was graded G3 and the remaining seven were graded G2. Distant metastases were absent in all patients. The patients’ medical records were reviewed for the following clinicopathological factors: age, gender, type of surgery, tobacco and alcohol use, tumor location (glottic/supraglottic), and follow-up status (Table S1).

### 2.2. Pathologic Evaluation of the Larynx Surgical Specimens

Surgical specimens of the larynx were examined and processed for histological examination at the Department of Pathology. All specimens were fixed in 4% phosphate-buffered formaldehyde and routinely processed for paraffin embedding after decalcification with 10% nitric acid (HNO_3_). Different tumor characteristics were evaluated, including the location (glottic, supraglottic, infraglottic, or transglottic), involved side (unilateral or crossing the midline), size, pattern of growth (exophytic or endophytic), ulceration, depth of invasion, spread to extralaringeal tissues, color, and features of the mucosa near the neoplasia. Data regarding the presence/absence of neo-plastic residual on resection margins was evaluated through ink application. Different sections of each tumor were sampled and collected so as to be representative of the maximum tumor invasion and of the interface between the tumor and adjacent normal mucosa. The pathological evaluation also included the characteristics of the lining epithelium of the mucosa adjacent to the tumor (e.g., metaplasia and dysplasia). All the lymph nodes that could be found from every specimen were also sampled for histological evaluation. Four-micrometer-thick tissue sections were cut from each paraffin block and used for hematoxylin–eosin staining (H&E; Figures S1–S3).

The H&E slides were imaged using the Olympus Slideview VS200 ASW for serial and automated histological acquisition. The preparation of manuscript figures was performed by using the QuPath open-source software for digital pathology and whole-slide image analysis (QuPath 0.3.0) [17]. Each specimen was evaluated for staging according to the AJCC, 8th Edition.

### 2.3. Immunofluorescence and Confocal Studies

Four surgical specimens of the larynx (Figure S1) were selected to be processed for Immunofluorescence (IF) analysis, including stage II (*n* = 1), stage III (*n* = 1), stage IVa (*n* = 1), and stage IVb (*n* = 1) LSCC samples. Antigen retrieval was achieved by using sodium citrate buffer (pH 6.0) for 30 min at 37 °C on deparaffined sections. The slides were then cooled to Room Temperature (RT) and washed three times with phosphate-buffered saline (PBS) solution. For autofluorescence quenching, the slides were incubated with ammonium chloride (50 µM, RT) for 30 min, and then blocked with 10% normal donkey serum (01700121, Jackson Lab, Bar Harbor, ME, USA). The overnight incubation (4 °C) with primary antibodies (anti-p75ICD 1:100; anti-p75NTR N-terminal, 1:100; and anti-ABCG2, 1:100) was followed by incubation with the appropriate combination of secondary antibodies. In particular, highly cross-adsorbed donkey anti-mouse-546 (A10036, Life Technologies, Carlsbad, CA, USA), donkey anti-rabbit-546 (A31572, Life Technologies, Carlsbad, CA, USA), and donkey anti-rat-488 (A21208, Life Technologies, Carlsbad, CA, USA) were used for double immunofluorescence (IF) (1:1000, 1 h, RT; Life Technologies, Carlsbad, CA, USA). Nuclei were counterstained with 1 µg/mL 4′,6-diamidino-2-phenylindole (DAPI; 1:1000; 15 min, RT; Life Technologies, Carlsbad, CA, USA).

Slides were coverslipped using ProLong Diamond mounting medium with anti-fading (P36931; Life Technologies, Carlsbad, CA, USA). Slide imaging was performed with the laser-scanning confocal microscope (Olympus FV1200) using a 20× air and a 40× (NA = 1.25) oil immersion lens, and visualized with FV10-ASW software (Version 4.2, Olympus). An UV diode laser operating at 405 nm, an Argon laser at 488 nm, and a HeNe laser at 543 nm were used as excitation sources. To entirely reconstruct the longitudinal tumor tissue, 14 confocal Z-stacks (1024 × 1024 pixels) were collected at 0.29 µm intervals in a 4 µm total optical depth, and then converted into max projection images. Omission of the primary antibody was routinely performed as a control for antibody specificity.

Images for direct comparison were collected using the same parameters and were analyzed with the help of FIJI open-source software (Image J; NIH, Bethesda, MD, USA).

### 2.4. Pathological Marker Positivity Scoring System

In order to compare the expressions of p75ICD and p75NTR in the resected specimens at different stages, they were evaluated by means of a semi-quantitative immunohistochemical assessment based on the intensity of immunoreactivity and its distribution [18,19]. The positivity was classified into three grades: “weak (+)”, “medium (++)”, and “strong (+++)”, and categorized as low (+) and high (++/+++).

### 2.5. Western Blotting

For Western Blot (WB) samples, 50 µg of protein was separated on precast 4–12% gel. At the end of electrophoresis, the proteins were stained with Ponceau Solution. Non-specific protein-binding sites on the nitrocellulose blots were blocked with PBS containing 7% non-fat milk (PBS–milk) for 1 h at RT. The primary antibodies were diluted in PBS at the following dilutions: 1:1000 for p75NTR C-terminal, 1:200 for ABCG2 (BXP-53: sc-58224), and 1:1000 for GAPDH antibodies. The incubation of the nitrocellulose blots with these antibodies was performed overnight (4 °C) at RT. The blots were then washed three times with PBS and incubated for 4 h at RT with the appropriate peroxidase-conjugated antibodies diluted 1:1000 in PBS–milk. Following PBS washes, the peroxidase activity of the nitrocellulose-bound secondary antibodies was detected with the Clarity West-ern ECL chemiluminescent reagents (BioRad Laboratories), imaged by Chemidoc XRS+ Image System (BioRad Laboratories), and bands were quantified by gel densitometry using the Fiji (ImageJ 1.53c) software (NIH, Bethesda, MD, USA). All the whole Western blot figures can be found in the Supplementary Materials.

## 3. Results

### 3.1. p75NTR Expression and p75ICD Generation by Regulated Cleavage in LSCC Tissue

**Figure 1:**
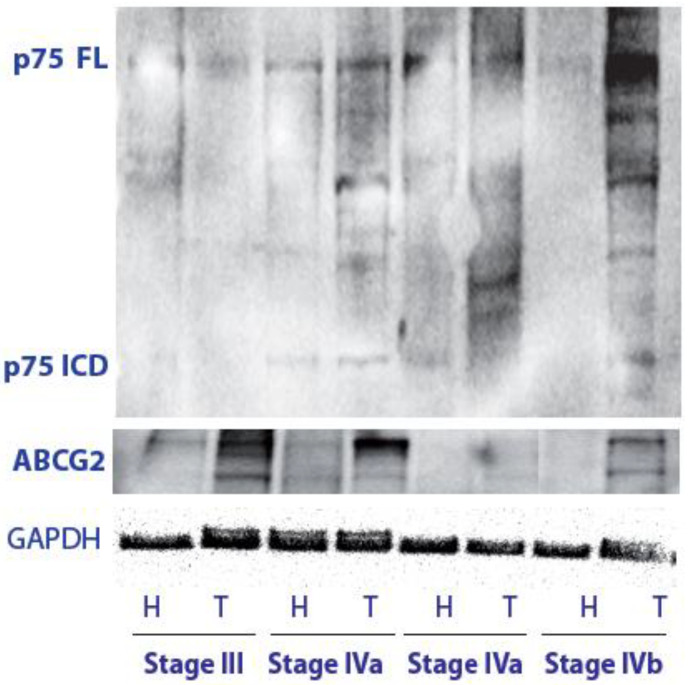
Representative WB of the p75NTR signaling molecule showing the full length and its cleavage pattern in stages III-IV LSCC. ABCG2 expression was also investigated to study the p75NTR and CSCs marker expression levels at different LSCC stages. GAPDH was used as a control protein for normalization. H = Healthy, T = Tumor. p75NTR = p75 Neurotrophin Receptor, p75NTR FL = p75NTR Full Length, p75ICD = p75NTR Intracellular Domain, ABCG2 = ATP Binding Cassette Subfamily G Member 2, and GAPDH = glyceraldehyde-3-phosphate dehydrogenase. Original blots in Figure S4.

We analyzed the p75NTR FL level and cleavage by using a C-terminal-specific anti-p75NTR antibody able to recognize both the p75ICD and the p75NTR FL by a biochemical assay. The p75NTR FL level was found to be detectable and high in all LSCC cases, while it was almost undetectable in the paratumoral tissue (Figure 1). Moreover, in addition to the p75NTR FL at 75 kDa, p75NTR cleavage fragments, and, in particular, p75ICD at 17–20 kDa, were also observed in tumor tissues, but not in the adjacent paratumoral samples.

The ABCG2 expression was also investigated in the same samples to compare the p75NTR and CSC marker expression levels in the different LSCC stages, and glyceraldehyde-3-phosphate dehydrogenase (GAPDH) was used as a control protein for normalization. ABCG2 is a family member of the ABC transporter, and its positive expression in laryngeal carcinoma has been related to tumor differentiation, age, sex, and presence of loco-regional lymph node metastasis. It has been previously suggested that the abnormal expression of ABCG2 may be related to the invasion and metastasis of laryngeal carcinoma, thus providing a good reference basis for the evaluation of LSCC invasivity [20].

The analysis of the tumoral marker in stages III and IV, with (on the left; T4N1, T4N3b) and without (on the right; IVa; T4N0) lymph nodes metastases, confirmed that LSCC cancer showed the maximal ABCG2 expression detectable by WB at stage III and further increased at stage IVa just before metastatization, being downregulated in the more advanced stages (IVb) as compared with previous tumoral stages.

Interestingly, initial p75ICD positivity could be observed at stage IVa, corresponding to the highest ABCG2 level. p75ICD was still high with an apparent slight increase at stage IVb of LSCC tumors.

### 3.2. Preliminary Characterization of the p75ICD and ABCG2 Immunofluorescent Staining Patterns in LSCC Tumor Samples

**Figure 2:**
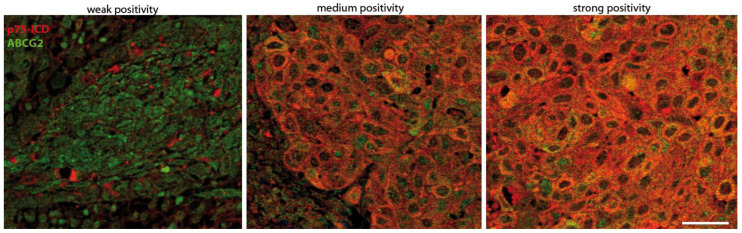
Representative immunostaining intensity for p75ICD expression scoring. Images exhibit weak (+), medium (++), and strong (+++) immunofluorescent labeling of p75ICD (red) and ABCG2 (green) in LSCC tissues. Scale bar: 100 µm.

As mentioned in Section 2, the p75ICD staining and its co-localization with the ABCG2 marker, were firstly identified in the LSCC tissues. p75ICD immunofluorescence staining was distinguished as weak (+), medium (++), and high (+++) immunofluorescence (Figure 2). As can be seen, p75ICD was found primarily in the cytoplasm of epithelial and cancer cells, although nuclear staining was also clearly detectable.

In line with the histological characteristics of LSCC tissue (Figure S2), p75ICD-positive cells showed an aberrant morphology with enlarged cell soma and nuclei, and most of them co-expressed the ABCG2 marker as shown in Section 3.3. Non-atypical epithelium produced weak positive p75ICD labeling.

### 3.3. p75ICD and ABCG2 Distribution in LSCC Tissue at Different Stages

**Figure 3:**
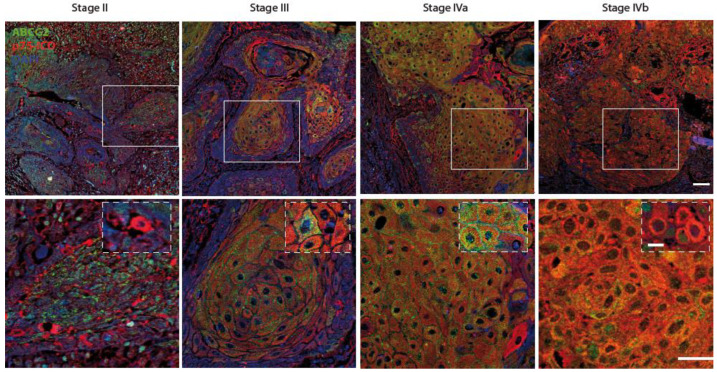
Representative p75ICD/ABCG2 co-localization in stage II-IVb LSCC specimens (*n* = 1 per clinical stage). Immunofluorescent labeling of p75ICD (red) and ABCG2 (green) at high magnification of specimens from stage II-IV LSCC patients. DAPI (blue) was used to counterstain nuclei. Scale bars: 50 µm; islets: 30 µm.

Figure 3 depicts the representative immunofluorescent staining of p75ICD (red) and ABCG2 (green) in the cancer stroma of LSCC patients from stages II, III, IVa, and IVb.

p75ICD was weak in stage II, while a progressive increase in the number of p75ICD-positive cells was noticeable from stages III to IVb, where a further increase in the single cell expression level was found in cells with enlarged nuclei, most of them also positive for the ABCG2 cancer marker. p75ICD was co-expressed by ABCG2-positive cells from stage II onward, while p75ICD/ABCG2 co-localization could not be detected in stage II stromal cells.

Indeed, the p75ICD staining could be cytosolic, as well as nuclear, while ABCG2 staining was mainly cytosolic and punctuate, in line with its expression in extracellular vesicles (Figure 3, high magnifications and islets).

**Figure 4:**
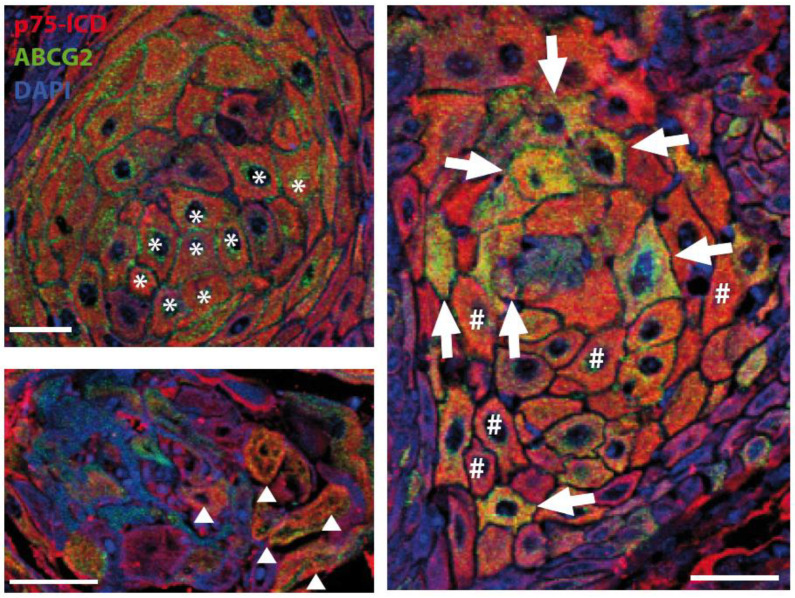
p75ICD expression in ABCG2-positive and negative cancer cells. Representative immuno-fluorescent labeling of p75ICD (red) and ABCG2 (green) at high magnification in specimens from stage IVa LSCC patients. DAPI (blue) was used to counterstain nuclei. Perinuclear ABCG2 expression (asterisk); weak (hashtag) and strong (arrow) cytosolic co-expression; shrunken cells undergoing the final stages of degeneration (triangle). Scale bars: 50 µm.

These differences are better appreciable in the high-magnification picture in Figure 4. Specifically, the co-localization of p75ICD and the marker ABCG2 was observed in invasive carcinoma CSCs, where ABCG2 staining was perinuclear within cells as well as pericellular (asterisks), as expected based on the ABCG2 expression in extracellular vesicles in squamous cancer. Moreover, p75ICD co-expression was found in cancer cells with weak (hashtag) and strong (arrow) ABCG2 expression in the cytosol. Notably, p75ICD was also found to be highly expressed in shrunken cells undergoing the final stages of degeneration (triangle).

In Table 2 below, the schematization of the semi-quantitative assessment of marker scoring indicating the p75ICD immunofluorescent intensity at LSCC stages II–IV is reported. 

### 3.4. Characterization of p75NTR and p75ICD Expressions in the Four Different Zones of LSCC Specimens

The distribution of cells expressing p75ICD and/or p75NTR FL, and their eventual co-localization with ABGC2, was also analyzed in each LSCC specimen by differentiating four distinct zones in the tumor sample, corresponding to: normal epithelium (1), aberrant epithelium (2), the carcinoma invasive front (3), and carcinoma in situ (4) (Figure S3).

**Figure 5:**
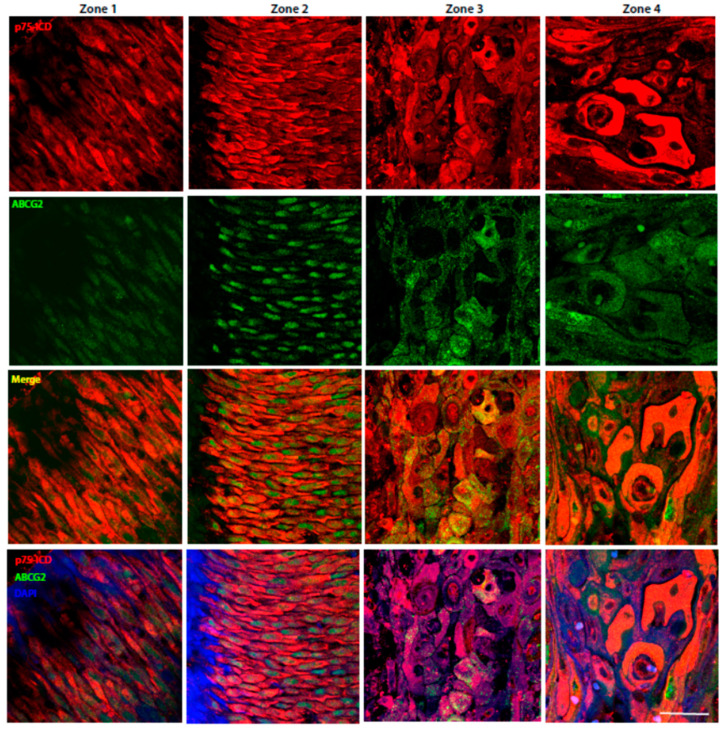
Representative p75ICD/ABCG2 colocalization analysis in the four zones, identified as in Figure S2. Immunofluorescent labeling of p75ICD (red) and ABCG2 (green) at high magnification in specimens from stage II-IV LSCC patients (*n* = 1 per clinical stage). DAPI (blue) was used to counterstain nuclei. Scale bar: 50 µm.

In Figure 5 are showed the representative pictures of p75ICD (red) and ABCG2 (green) immunofluorescent staining of Zones 1–4 from a stage III LSCC patient. The p75ICD positivity was weak and mainly cytosolic in most of the non-atypical epithelial cells of Zone 1, where ABCG2 staining was almost undetectable under our experimental conditions. Prevalent nuclear ABCG2 staining was evident in dysplastic epithelial cells of Zone 2, where p75ICD staining was more diffuse and mainly cytosolic.

As reported in Zones 3–4, p75ICD expression was prominent in cancer cells with a highly dysmorphic appearance and enlarged nuclei, and was more evident in Zone 4 as compared with Zone 3.

Note that not all of the p75ICD positive cells with aberrant morphology also expressed ABCG2.

**Figure 6:**
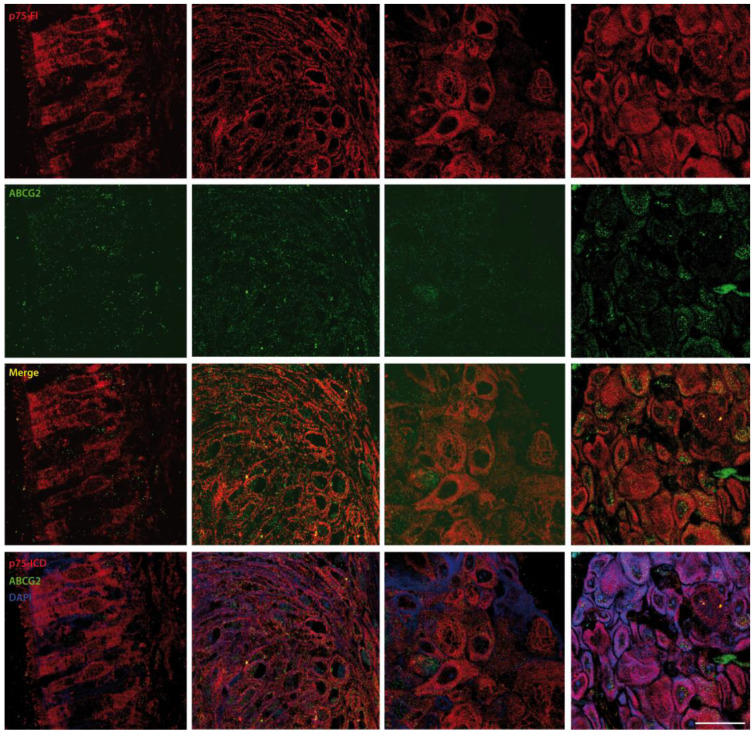
Representative p75NTR/ABCG2 co-localization analysis in the four zones identified as in Figure S2. Immunofluorescent labeling of p75NTR (red) and ABCG2 (green) at high magnification in a specimen from stage II-IV LSCC patients (*n* = 1 per clinical stage). DAPI (blue) was used to counterstain nuclei. The semi-quantitative evaluation of the distribution of the p75ICD, p75NTR, and ABCG2 cells in the diverse zones is shown in Table 3 below. Scale bar: 50 µm.

Representative pictures of the co-localization of the p75NTR (red) and ABCG2 (green) in Zones 1–4 from a stage III LSCC patient are shown in Figure 6. The p75NTR expression in normal elongated epithelial cells was weak and cytosolic, as observed in Zone 1. The expression of p75NTR was prominent in the atypical epithelial cells of Zone 2, and its expression appeared to be significantly upregulated in some shrunken cells with enlarged nuclei in Zones 3–4, with the highest expression in the latter. Please note that the p75NTR antibody used for IF was directed against its N-terminal, excluding the possibility of recognizing the C-terminally cleaved p75ICD fragment.

### 3.5. Differential Intracellular Localization of p75NTR FL and p75ICD in LSCC

**Figure 7:**
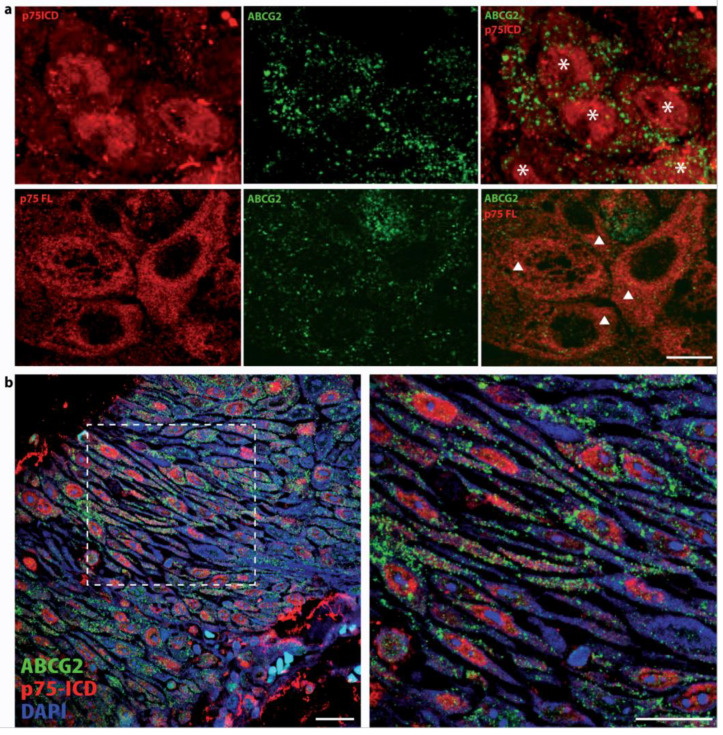
(**a**) p75ICD versus p75NTR FL intracellular immunostaining. Representative immunofluorescent labeling of p75NTR FL (red) and ABCG2 (green) in the nuclei (asterisks), and cytosol (triangle), respectively, from the stage IVb LSCC specimen. Scale bar = 20 µm. (**b**) Representative p75ICD/ABCG2 co-localization analysis in the abnormal epithelium of a LSCC specimen. Immunofluorescent labeling of p75ICD (red) and ABCG2 (green) at high magnification in epithelium from stage II LSCC. DAPI (blue) was used to counterstain nuclei. Scale bars = 50 µm.

p75NTR FL and its C-terminal fragment p75ICD showed not only differential expression levels, but also distinctive intracellular localization. In fact, while the p75NTR FL was found to be mainly cytosolic (Figure 7a), the p75ICD could be both cytosolic and nuclear, with the nuclear localization being prevalent in epithelial cells prior to dysplastic progression (Figure 7b) and in both ABCG2-positive and negative cancer cells (Figure 7a), with a clear dysmorphism and enlarged nuclei. 

Interestingly, the p75ICD staining was also found to be nuclear in stage II epithelial cells (Figure 7b).

## 4. Discussion

The present pilot study investigated the occurrence and, eventually, the pathological significance of the p75NTR-regulated cleavage by α- and γ-secretases, and the consequent release of the intracellular p75ICD fragment in LSCC. In particular, the study addressed the question of whether the distribution of p75ICD fragments might differ from the p75NTR FL, the expression of which has been largely described in several cancer types [13,14,16], and characterized their respective expressions in CSCs.

NGF and its receptors (TrkA and p75NTR) are implicated in cancer growth, CSC proliferation, and immune system evasion [21]. p75NTR has been shown to exert anti-apoptotic functions, possibly upon stimulation by the precursor form of NGF (proNGF), in tumor growth and spreading, which is largely independent of the mitogenic NGF/TrkA pathway [22]. Despite preliminary indications regarding the role of p75NTR maturation in cancer progression, the control of p75ICD generation by regulated cleavage and its expression and distribution in LSCC of relevance for cancer progression and metastatization have not been addressed so far. In particular, direct proof of the regulated cleavage of p75NTR leading to the generation of p75ICD in cancer cells is still lacking in most malignant tumors, including LSCC.

Here, we used the specific anti-p75ICD antibody that does not cross react with the p75NTR FL molecule to investigate its expression and localization in primitive LSCC and its nodal metastasis in a small group of patients representative of the diverse stages of LSCC. 

Furthermore, since p75NTR is also present in CSCs and is co-expressed with stemness markers, such as CD133 in LSCC [16], and with ABCG2 in tumors involving different organs, and also in primitive hepatic and prostatic cancers [23], the possibility that p75NTR might be a novel CSC marker of LSCC has also been explored. The expression and localization of ABCG2, which has been associated with invasion, metastasis, and resistance to chemotherapy in squamous carcinoma and laryngeal carcinoma [24] and is an unfavorable prognostic factor in HNSCC [23], was sorted by us to investigate the cell-type-specific localization of p75NTR and p75ICD in CSCs in LSCC.

To resolve this lack of information, we first asked whether p75NTR undergoes regulated cleavage also in squamous cancers, such as LSCC, as it has been extensively reported in brain neurodegeneration and gliomas [3]. We found an increase in the p75ICD level at stage IV, just before the lymph node involvement stage, and it was still high in metastatic LSCC (Figure 3), while the level of p75NTR FL was found to be decreased as compared with LSCC stage III. Of note, and in line with the literature, the p75NTR level rose in advanced LSCC, when, in turn, p75ICD was almost undetectable under our experimental conditions (Stage IVb). Although this finding needs to be consolidated by a larger patient study, it indicates that the generation of p75NTR fragments is an important element for investigating cancer progression.

In fact, most of the findings reported by the literature do not allow discriminating between the two forms of the p75NTR, lacking the critical knowledge to target the p75NTR signaling for LSCC therapy. A deeper understanding of the opposite p75NTR and p75ICD-mediated biological functions could also help in explaining some apparently contradictory effects of p75NTR in cancers. In fact, behind the cancer-specific developmental mechanisms, p75NTR has been reported to be pro-apoptotic in gastric cancer [14] and proliferative in tongue [9] and esophageal squamous carcinogenesis [6].

In order to specifically investigate p75ICD’s biological action by immunofluorescent labeling in LSCC specimens, we resorted to a validated and specific anti-p75ICD antibody [25], and a commercial antibody directed against the N-terminal of p75NTR, recognizing the entire p75NTR molecule.

Thus, we performed double IF staining for p75NTR/ABCG2 and p75ICD/ABCG2, and scored immunopositivity grading by the semiquantitative assessment method based on weak (+), medium (++), and strong (+++) immunolabeling (Figure 2).

First of all, we observed that the two molecules had distinct patterns of expression (Figure 5 and Figure 6) in the four different zones (as identified in Figure S3). Indeed, the immunofluorescence findings were in line with the biochemical analysis, pinpointing a higher level of p75NTR in LSCC stages II and IVb, where the p75NTR expression was also found to be maximal by WB analysis (Figure 1).

Then, we investigated p75ICD expression at different LSCC stages (II-IV) and found a progressive increase in the number and intensity of p75ICD-immunolabeled cells from stage II toward stage IV (Figure 3; Table 2), with few cells positive to p75ICD in LSCC stage II, reaching a maximal diffuse expression in LSCC stage IVb.

Additionally, we explored p75ICD expression and localization in the four different zones of the single LSCC tumor specimen (Figure 5; Table 3) and found that p75ICD was present at a low level in the cytosol of the non-atypical epithelium, slightly increasing in dysplastic epithelial cells (Figure 5, Zone 2) and in the front of invasion (Figure 5, Zone 3) to reach the highest level of expression in late-stage invasive carcinoma cells, mainly in highly dysmorphic cells with a polygonal appearance.

Additionally, we studied the p75NTR level and distribution in adjacent sections and found that p75NTR expression increased in aberrant (Figure 6, Zone 2) as compared with normal (Figure 6, Zone 1) epithelia, and it was still high in stromal LSCC cells of invasive carcinoma (Figure 6, Zone 4), while it underwent an apparent reduction in the front of invasion (Figure 6, Zone 3). These latter observations are in line with the literature in the field, showing that p75NTR expression is promoted in epithelial stem cells and in moderate to severe dysplasia, but is specifically downregulated at the melanoma invasion front [26,27].

These observations are partially in line with the study by Li and collaborators, in which the immunohistochemical localization of p75NTR was reported in normal, paratumoral mucosa, and LSCC without any significant difference in the p75NTR level between dysplastic epithelia and LSCC. Unlike the specific antibodies used in this study and directed against the extracellular domain (p75NTR FL) and, specifically, the ICD domain (p75ICD) of the p75NTR, Li’s study reported data obtained using an anti p75NTR C-terminal antibody, which virtually recognized p75NTR and all of its fragments, including the p75ICD (although minor expected affinity). This fact might account for the difference in the trend and distribution, and also for the lack of correlation between p75NTR expression, histopathologic grading, and lymph node metastases. The same authors observed that “with the deterioration of differentiation, the ratio of p75NTR-positive cell increased partly, but the coloration intensity of each tumor cell degraded, with very faint cytoplasm coloration of p75NTR” [16]. Since we found that p75NTR FL is mainly cytosolic (Figure 7a, lower picture), while p75ICD nuclear localization is prevalent in epithelial cells prior to dysplastic progression (Figure 7b), it is likely that the use of specific p75NTR antibodies is crucial to determine the single specific contribution of p75NTR and p75NTR fragments in cancer tissues and their metastatic progression.

In accordance, we found that the p75ICD pattern of expression in the different LSCC zones correlated with ABCG2 expression better than the p75NTR profile, as reported in Table 2 and Table 3. Indeed, the expression pattern observed by WB also suggested p75ICD as a better correlation of ABCG2 than its full length form, at least in the first stages of pathology (Figure 1).

Furthermore, p75ICD vastly co-localized with ABCG2-positive cells, some expressing low-intensity ABCG2 immunolabeling, and others morphologically abnormal with both intracellular and extracellular ABCG2 staining (Figure 4). Note that p75ICD was also expressed in shrunken degenerating cells (Figure 4, triangle).

Based on the critical role of p75ICD nuclear translocation for its functions in the central nervous system, the subcellular localization of p75ICD (cytosolic versus nuclear) was also assessed in the resected specimen from LSCC stages II-IV. In agreement with the augmented nuclear p75ICD staining reported in neurodegenerating neurons of Alzheimer’s Disease patients, we found prevalent p75ICD nuclear localization in aberrant epithelia from LSCC tumor stage II (Figure 7b), as well as in CSCs of invasive LSCC stage IVb (Figure 7a).

The p75NTR has been demonstrated to be a central regulator of cancer invasion in highly invasive glioma [3], and its proteolysis has been supposed to be a strict requirement for glioma invasion [28]. Moreover, p75NTR-overexpressing cells have also been shown to confer migratory and invasive potential to cancer cells in vitro [29]. p75NTR is expressed at the invasive front of the tumor, suggesting that p75NTR-positive cells may be invasive cancer cells [30]. In this context, it is relevant that the perineural release of NGF promotes CSCs proliferation and the initiation of the pro-oncogenic pathway [22]. Additionally, the intratumoral perineural invasion, which predicts patients at high risk of being diagnosed with lymph-node metastases, has been recently proposed as an additional prognostic factor in HNSCC [31].

Therefore, our study showing that the p75NTR and expression patterns differ in a stage-specific and zone-related manner, and that p75ICD is co-localized with the metastatic marker ABCG2 and shows a prevalent nuclear expression in aberrant and highly dysmorphic cancer cells, led us propose p75ICD as a good candidate prognostic factor in LSCC. Moreover, the presence of p75ICD in ABCG2-positive cancer cells surrounded by putative ABCG2 positive vesicles (as visible in Figure 3, Figure 4 and Figure 7a) is a good foundation for further analysis of p75ICD implication in exosome-driven cancer drug resistance.

Larger studies or meta-analyses will eventually help to reach sufficient statistical power to assess the utility of p75ICD as a prognostic factor in LSCC.

Of note, p75NTR expression has been suggested by others to be prognostic when in combination with the marker CD44 in esophageal, hypopharyngeal, and oral carcinomas, as well as in HNSCC [10]. Despite the obvious limitations due to the limited number of LSCC specimens and patients included in the study, the intriguing preliminary findings described here open the way for a systematic analysis of stage and cell-type-specific p75ICD expression in the progression and invasion of LSCC, as well as other non-neuronal and squamous cancers.

## 5. Conclusions

An in-depth study and revelation of the molecular mechanisms controlling normal epithelia and those events underlying its transformation toward metastatic SCC is of foremost importance in the quest for novel biomarkers improving early diagnosis and personalized formulation in the clinical treatment of LSCC.

Although these results arose from a pilot study, and therefore need to be further explored in a larger patient group, our findings indicate that p75ICD generation by p75NTR-regulated cleavage might represent a relevant marker of tumor invasion and metastasis in LSCC, also supporting a role for p75NTR/p75ICD in the regulation of CSC proliferation.

p75NTR was implicated in the control of the apoptotic machinery in neuronal cells and was indicated as a good pathological marker for the progression and spreading of both central nervous system cancers and non-neuronal carcinoma, such as LSCC. Although the exact mechanism is not clear, it is highly likely that p75NTR may predispose tumor cells to perineural invasion, conferring responsivity to NGF-producing Schwann cells and neurons, as reported in oral cancer and pancreatic cancer [32,33]. Further, p75NTR may allow cells to survive hypoxia by stimulating hypoxia-inducible factor 1a (HIF-1a) and Vascular Endothelial Growth Factor (VEGF)-dependent neo-angiogenesis [34]. Last, but not least, the presence of p75ICD (but not p75NTR FL) in CSCs surrounded by what is reminiscent of ABCG2-positive extracellular vesicles suggests a role for p75ICD in drug resistance, a major factor limiting the efficacy of chemotherapy in patients with laryngeal cancer.

Overall, it is advisable to foster our knowledge on p75NTR cleavage control at different stages of tumor development and specifically investigate p75ICD as a novel prognostic factor possibly improving diagnosis and instructing personalized treatment in LSCC, as well as other squamous cancers.

## Figures and Tables

**Table 1:** Pathological TNM (pTNM) stages, American Joint Committee on Cancer (AJCC) stages, and Grading (G) of Laryngeal Squamous Cell Carcinoma (LSCC) patients.

pTNM Stage	AJCC Stage	G
pT3N0M0	III	G2
pT4aN0M0	IVa	G2
pT3N3bM0	IVb	G2
pT4aN3bM0	IVb	G3
pT4aN0M0	IVa	G2
pT4aN1M0	IVa	G2
pT2NXM0	II	G2
pT4aNXM0	IVa	G2

**Table 2:** Semi-quantitative scoring of p75ICD (p75NTR Intracellular Domain) and ABCG2 (ATP Binding Cassette Subfamily G Member 2) immunopositivity in different LSCC (Larynx Squamous Cell Carcinoma) stage specimens (*n* = 1 per clinical stage). Positivity was classified with three grades; “weak (+)”, “medium (++)”, and “strong (+++)”, according to the scoring references reported in Figure 2.

Stage	p75ICD	ABCG2
II	+	++
III	++	++
IVa	++	+++
IVb	+++	+

**Table 3:** Semi-quantitative scoring of p75ICD (p75NTR Intracellular Domain), p75NTR FL (p75 Neurotrophin Receptor Full Length), and ABCG2 (ATP Binding Cassette Subfamily G Member 2) immunostaining in the 4 different zones of a LSCC specimen at stage III. Positivity was classified into three grades: “weak (+)”, “medium (++)”, and “strong (+++)”.

LSCC	p75ICD	p75NTR FL	ABCG2
Zone 1	+	++	+
Zone 2	++	+++	++
Zone 3	++	+	+++
Zone 4	+++	++	++

## Data Availability

Data are contained within the article or Supplementary Material. All data generated or analyzed during this study are included in this published article (and its Supplementary Information files).

## Supplementary Materials

The following supporting information can be downloaded at: https://www.mdpi.com/article/10.3390/cancers14112622/s1, Table S1: Information on patient gender, age, type of surgery, tobacco and alcohol use, tumour location and follow up status; Figure S1: Representative pictures of tumour resected samples at different stadiations (II-IVb). Figure S2: LSCC carcinoma histological hallmarks. Figure S3: Haematoxylin & Eosin (H&)E low magnification staining of a LSCC tumour specimen stage III. Figure S4: Original blots.
