# Supplementary material for: Pilot Investigation on p75ICD Expression in Laryngeal Squamous Cell Carcinoma
Source: Cancers (Basel). 2022 May 25;14(11):2622. doi: 10.3390/cancers14112622 (PMC9179539; doi:10.3390/cancers14112622)
Supplement: Supplementary file 1 [file cancers-14-02622-s001.zip › cancers-1704557-supplementary.pdf]

Supplementary data

1. Patients

Information on patient gender, age, type of surgery, tobacco and alcohol use, tumour location and follow up status are also shown (Tables S1). A value of '0' indicates no or infrequent use of tobacco or alcohol products, as reported by patients; a value of '1' indicates self-reported regular, heavy use of tobacco or alcohol products (Table S1).

The study was performed on a total of 8 patients (seven males and 1 female). The mean age was 72.9 years (range 61-79); half of the patients referred alcohol consumption, while 7 reported themselves as heavy smokers. No previous familiar case of LSCC was reported in the anamnesis.

The main site of tumor location is the glottis (seven cases) and in only one case the cancer arises from the supraglottic laryngeal area.

The mean follow-up was 9 (8.75) months. At the end of follow-up we reported 1 patient died for other cause (DOC) and 7 cases were free of disease (NED, No Evidence of Disease).

Table S1.

SEX/AGE	SURGERY	TOBACCO/ALCOHOL USE	TUMOR LOCATION/SUBSITES	FOLLOW UP (MONTHS/STATUS)
M/79	OPHL 2B + LSND (II-IV)	1/0	Glottic/VOCAL CORD	12/NED
M/79	OPHL 1B + RSND (I-IV)	0/1	Glottic/RIGHT VOCAL CORD	12/NED
M/79	TL + RLND	1/1	Glottic/LEFT VOCAL CORD	9/NED
M/62	HYPOPHARYNGOLT + LSND (II-IV)	1/1	Glottic/LEFT VOCAL CORD	9/NED
M/77	TL + BSND (I-IV)	1/0	Glottic/ANTERIOR COMMISSURE	1/DOC
M/61	OPHL 2B + BSND (I-IV)	1/1	Glottic/LEFT VOCAL CORD	9/NED
M/72	OPHL 2A	1/0	Supraglottic/INFRAHYOID EPIGLOTTIS	9/NED
F/74	TL + BSND (II-IV)	1/0	Glottic/RIGHT VOCAL CORD	9/NED

TL: total laryngectomy, **RLND:** Radical left neck dissection, **BSND:** bilateral selective neck dissection, **RSND:** right selective neck dissection, **LSND:** left selective neck dissection, **OPHL:** open partial orizontal laryngectomy, **HYPOPHARYNGOLT:** hypopharyngolaryngectomy. **NED:** non evidence of disease, **DOC:** died for other cause

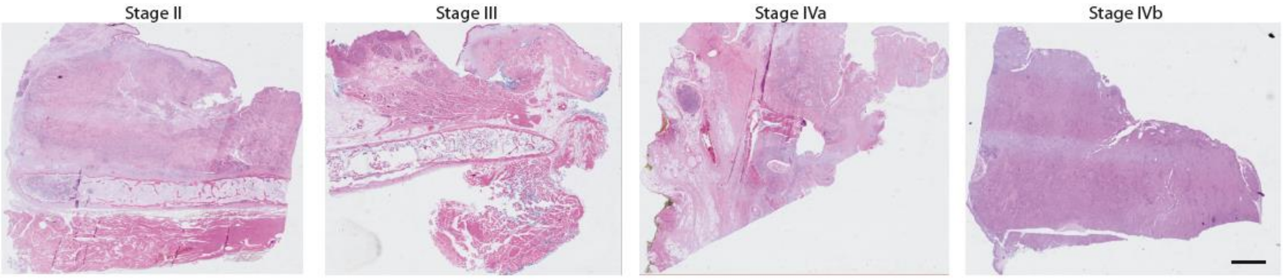

Figure S1. Representative pictures of tumour resected samples at different stadiations (II-IVb). Scale bar = 1mm.

2. Histopathological analysis for patient diagnosis and tumour stadiation.

The Figure S2 shows the high magnification of representative H&E staining of LSCC resected samples. H&E staining was also used as reference for the characterization of immunofluorescence staining of the pathological markers under investigations, namely p75ICD, p75NTR FL, ABCG2. The representative high magnification images in the panel of Figure S3 show typical histological hallmarks of laryngeal carcinoma, including clustered small nests and islands of neoplastic cells under keratinization. Also, cells with enlarged nuclei and abnormal nuclear morphology and enriched nuclear contents, as well as highly disordered cells with almost disrupted cell morphologies are clearly visible in LSCC invasive carcinoma.

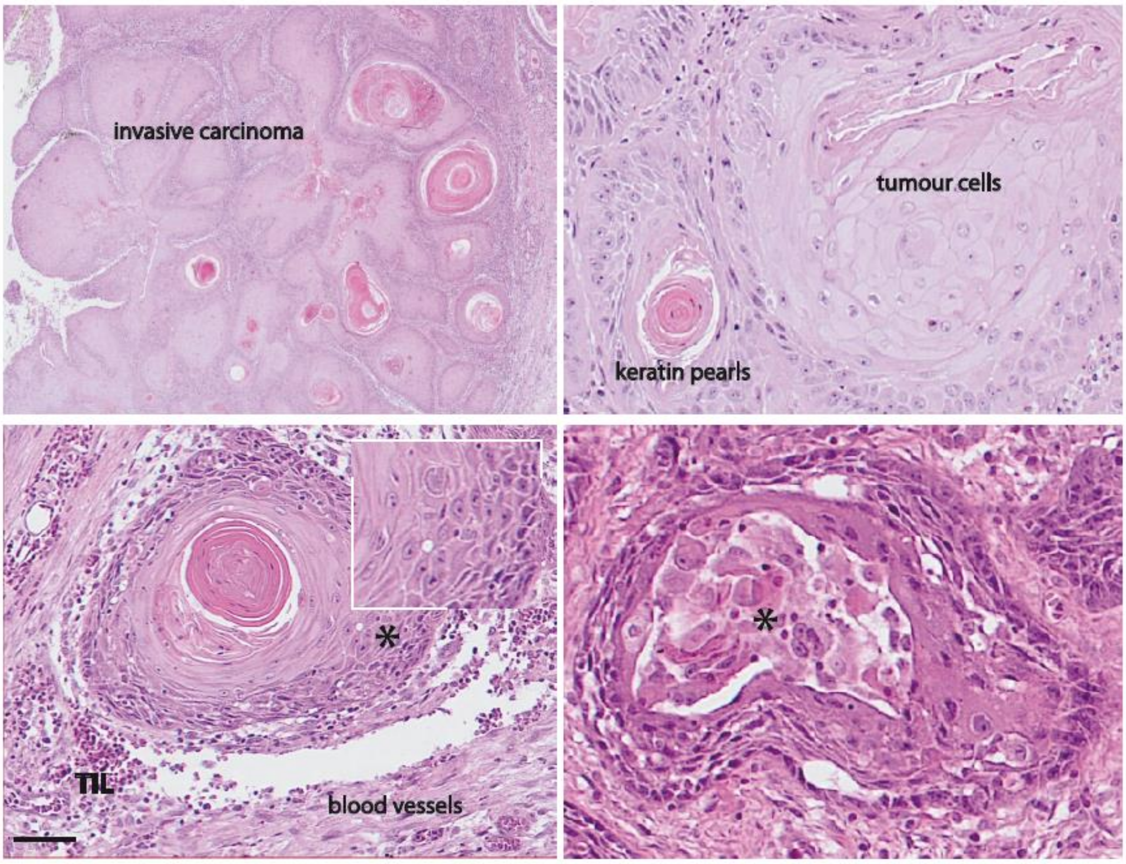

Figure S2. LSCC carcinoma histological hallmarks. Representative high magnification images of LSCC carcinoma showing invasive carcinoma below the lamina propria, tumour cells nests including shrinked tumour cells, tumour infiltrating lymphocytes (TIL), and abundant stromal blood vessels. Scale bar = 50μm.

3. Identification of four histopathological zones for the characterization and distribution analysis of p75NTR FL and p75ICD fragment.

Four zones were also identified for further IF analysis by resorting to H&E histological staining of resected cancer specimen: Zone 1, corresponding to non-atypical, normal epithelium; Zone 2, aberrant epithelium showing increased thickness and aberrant epithelial cells of the LSCC mucosa; Zone 3, the invasive front of LSCC carcinoma trespassing the basal lamina and entering the laryngeal stroma; Zone 4, carcinoma *in situ*, with multiple nests of cancer cells in the laryngeal stroma. An example is showed in Fig S3.

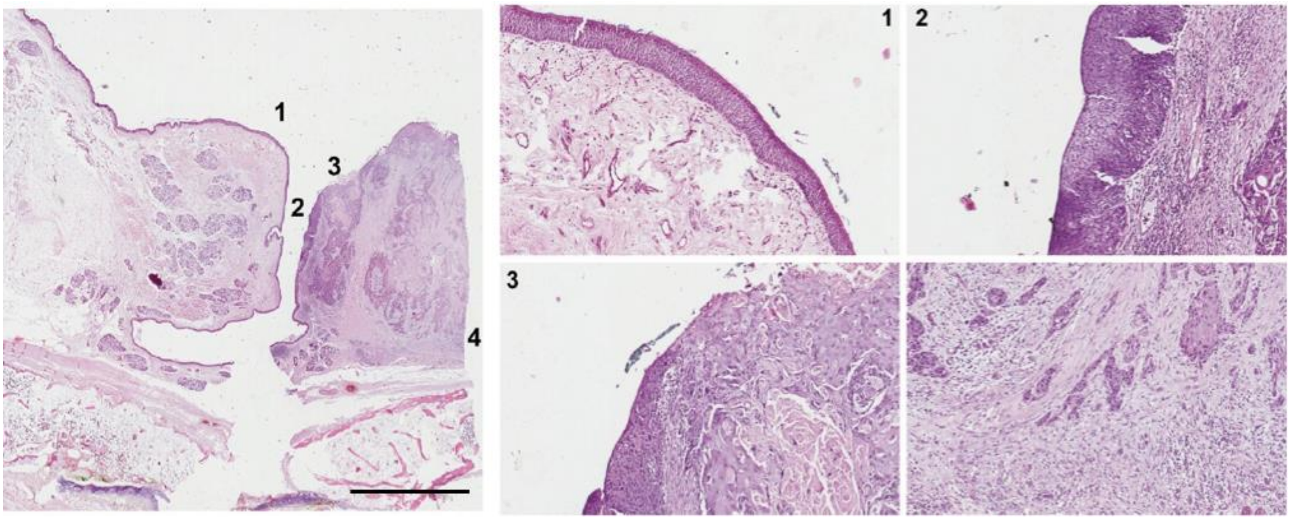

Figure S3. Haematoxylin & Eosin (H&)E low magnification staining of a LSCC tumour specimen stage III. Different zones at higher magnification are indicated, corresponding to: 1, non-atypical epithelium; 2, aberrant epithelium; 3, invasive carcinoma; 4, carcinoma in situ. Scale bar: 1 mm.
