# Supplementary figures and images for: Pilot Investigation on p75ICD Expression in Laryngeal Squamous Cell Carcinoma
Source: Cancers (Basel). 2022 May 25;14(11):2622. doi: 10.3390/cancers14112622 (PMC9179539; doi:10.3390/cancers14112622)

Blot: Anti-p75 Cterminal Ab

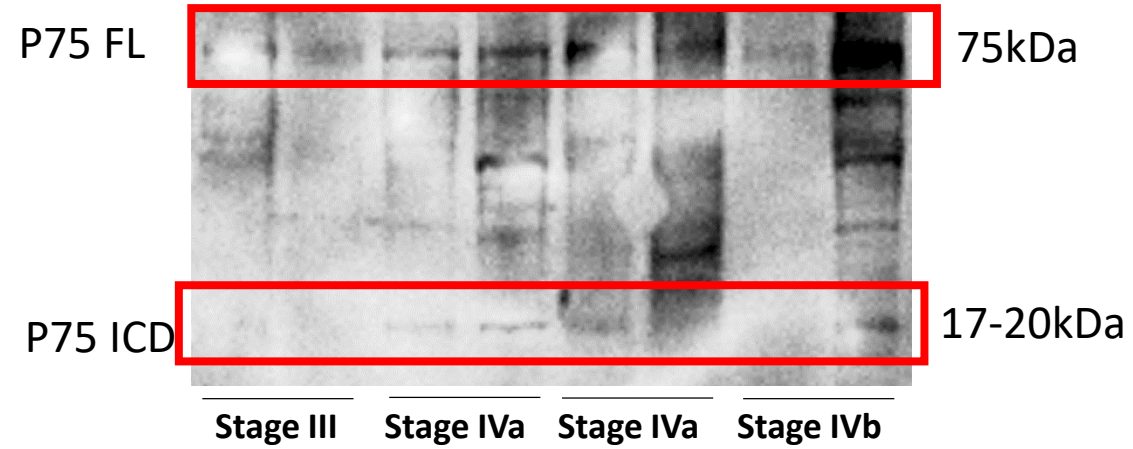

Blot: Anti-ABCG2 Ab

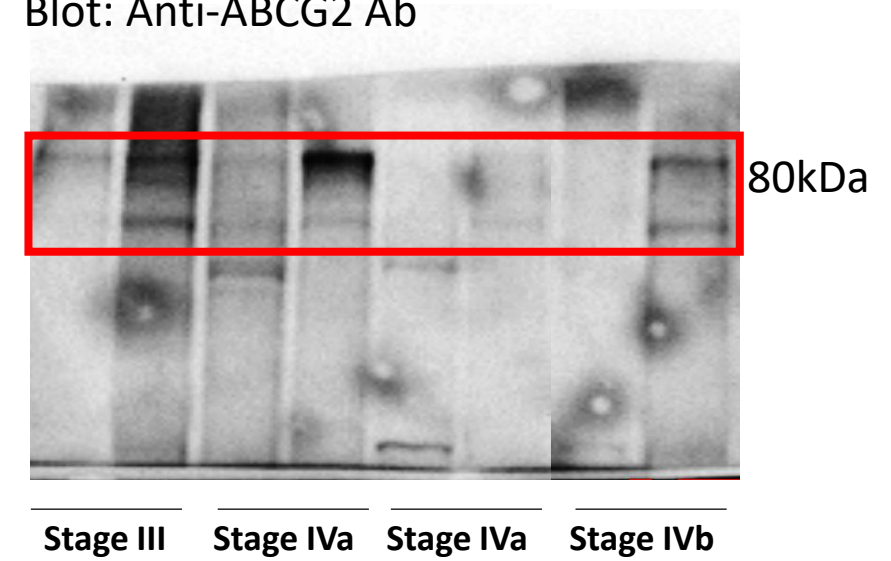

Blot: Anti-GAPDH Ab

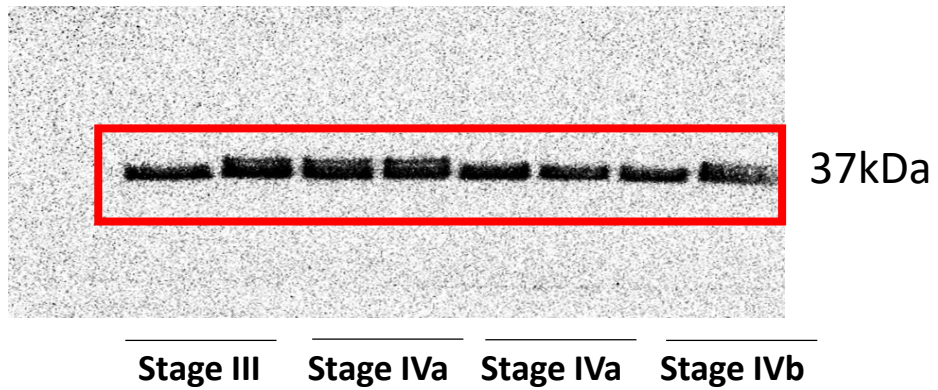

Supplement: Supplementary file 1 [file cancers-14-02622-s001.zip › cancers-1704557-Figure S4-original-images.pdf]
